# Deep-Water Chemosynthetic Ecosystem Research during the Census of Marine Life Decade and Beyond: A Proposed Deep-Ocean Road Map

**DOI:** 10.1371/journal.pone.0023259

**Published:** 2011-08-04

**Authors:** Christopher R. German, Eva Ramirez-Llodra, Maria C. Baker, Paul A. Tyler, and the ChEss Scientific Steering Committee

**Affiliations:** 1 Woods Hole Oceanographic Institution, Woods Hole, Massachusetts, United States of America; 2 Institut de Ciències del Mar, Consejo Superior de Investigaciones Científicas, Barcelona, Spain; 3 School of Ocean and Earth Science, University of Southampton, National Oceanography Centre, Southampton, United Kingdom; Université Paris Sud, France

## Abstract

The ChEss project of the Census of Marine Life (2002–2010) helped foster internationally-coordinated studies worldwide focusing on exploration for, and characterization of new deep-sea chemosynthetic ecosystem sites. This work has advanced our understanding of the nature and factors controlling the biogeography and biodiversity of these ecosystems in four geographic locations: the Atlantic Equatorial Belt (AEB), the New Zealand region, the Arctic and Antarctic and the SE Pacific off Chile. In the AEB, major discoveries include hydrothermal seeps on the Costa Rica margin, deepest vents found on the Mid-Cayman Rise and the hottest vents found on the Southern Mid-Atlantic Ridge. It was also shown that the major fracture zones on the MAR do not create barriers for the dispersal but may act as trans-Atlantic conduits for larvae. In New Zealand, investigations of a newly found large cold-seep area suggest that this region may be a new biogeographic province. In the Arctic, the newly discovered sites on the Mohns Ridge (71°N) showed extensive mats of sulfur-oxidisng bacteria, but only one gastropod potentially bears chemosynthetic symbionts, while cold seeps on the Haakon Mossby Mud Volcano (72°N) are dominated by siboglinid worms. In the Antarctic region, the first hydrothermal vents south of the Polar Front were located and biological results indicate that they may represent a new biogeographic province. The recent exploration of the South Pacific region has provided evidence for a sediment hosted hydrothermal source near a methane-rich cold-seep area. Based on our 8 years of investigations of deep-water chemosynthetic ecosystems worldwide, we suggest highest priorities for future research: (i) continued exploration of the deep-ocean ridge-crest; (ii) increased focus on anthropogenic impacts; (iii) concerted effort to coordinate a major investigation of the deep South Pacific Ocean – the largest contiguous habitat for life within Earth's biosphere, but also the world's least investigated deep-ocean basin.

## Introduction

The recognition that lush communities of endemic fauna were associated with seafloor venting systems when the latter were first discovered in the late 1970s [1] represents one of the major highlights of the past Century across all fields of scientific research. The deep seafloor had long been considered, in general, as being food poor and devoid of any *in situ* primary productivity in the absence of sunlight, hosting only low levels of secondary productivity and biomass [2]. While the potential for chemosynthesis (in which microbes live on energy released from purely inorganic chemical reactions) had been proposed as long ago as 1890 by Sergei Nikolaevich Vinogradskii, it was nearly a century later before such systems were demonstrated to be active on the floor of Earth's deep oceans [3].

Following the discoveries at the Galapagos vents in 1977, it was recognized that chemosynthetic ecosystems could also be sustained in other chemically reducing seafloor environments, such as at cold seeps along passive and active margins [4] and in association with large organic falls such as wood falls [5], whale skeletons [6]–[7] and, following pioneering work by Gallardo et al. [8], even in oxygen minimum zones (reviewed in [9]). In all cases, wherever there are chemically reduced compounds present (typically one or both of CH_4_ and H_2_S are most important) so, too, microbial activity is enhanced. These microbiota may be present in free-living form, in the deep water column or at the seafloor as microbial mats, within sediments or even within the fractures of crustal rocks, sub-seabed, or in symbiosis with larger multi-cellular organisms [10]. In all instances, these microbes mediate the transformation of chemical energy, to facilitate the development of densely populated ecosystems in which both faunal abundances and biomass are very much greater than is typical at the deep seafloor.

In addition to high concentrations of chemically reduced compounds, equally important to chemosynthetic ecosystems are the steep gradients that arise (both chemical and, in the case of hydrothermal vents, thermal) and the great propensity for geologic (tectonic and/or volcanic) disturbance that can initiate, perturb or curtail fluid flow from the seafloor. What is commonly observed among fauna colonizing vent- or seep-sites, consequently, is a low diversity of complex multi-cellular organisms despite the high biomass present [11]–[14]. Typically, approximately 50% of the species present are extremely rare, represented by no more than 5 individuals in collections of tens of thousands of specimens [15]–[16]. Deep-water chemosynthetic ecosystems also show high levels of endemicity within vent and seep habitats: 70% in hydrothermal vents [17] and ∼40% at seeps [18]–[19], whereas endemicity in OMZs, although not formally quantified, is considered to be relatively low [19]. Because chemosynthetic habitats are both ephemeral and are sparsely distributed across the seafloor, successful species must be specially adapted for both dispersal away from, and colonization of these discrete deep-ocean “oases”, in addition to exhibiting specific chemosynthetic adaptations [20]–[21].

Since the first discovery of vents in 1977, some 700 hydrothermal vent species previously unknown to science, together with 600 species at cold seeps, have now been described and listed in the ChEss database, *ChEssBase* [22]: www.noc.soton.ac.yj/chess/database/db_home.php. Most of the species described so far belong to the mega- and macrofauna size classes and, although the meiofauna accounts for 20% of the total diversity in vents [23], many species are awaiting discovery and description [14], [24]. The rate of species discovery from deep-water chemosynthetic sites equates, on average, to at least one new species being discovered and described every two weeks throughout the past 34 years. Yet, for vents, less than 20% of Earth's entire ridge-crest has been explored for hydrothermal activity. While more than 200 individual vent, seep and organic-fall chemosynthetic sites have now been visited and examined in at least some detail using deep-submergence vehicles, there are probably an order of magnitude more sites waiting to be discovered (see later).

## Methods

At the onset of the ChEss project, and despite the sparse global-scale coverage available then, it was widely accepted that at least 6 separate biogeographic provinces could already be shown to exist for hydrothermal vent fauna [25], whereas seep and whale-fall communities appeared to share many key taxa across all oceans [26]. This led to the identification of a series of inter-related problems that the ChEss project sought to address:

- Establishing phylogeographic relationships among different chemosynthetic habitats.
- Seeking evidence for barriers to, or conduits for, gene flow between different chemosynthetic habitats.
- Investigating the environmental factors that control the diversity and distribution of chemosynthetic fauna.

**Figure 1:**
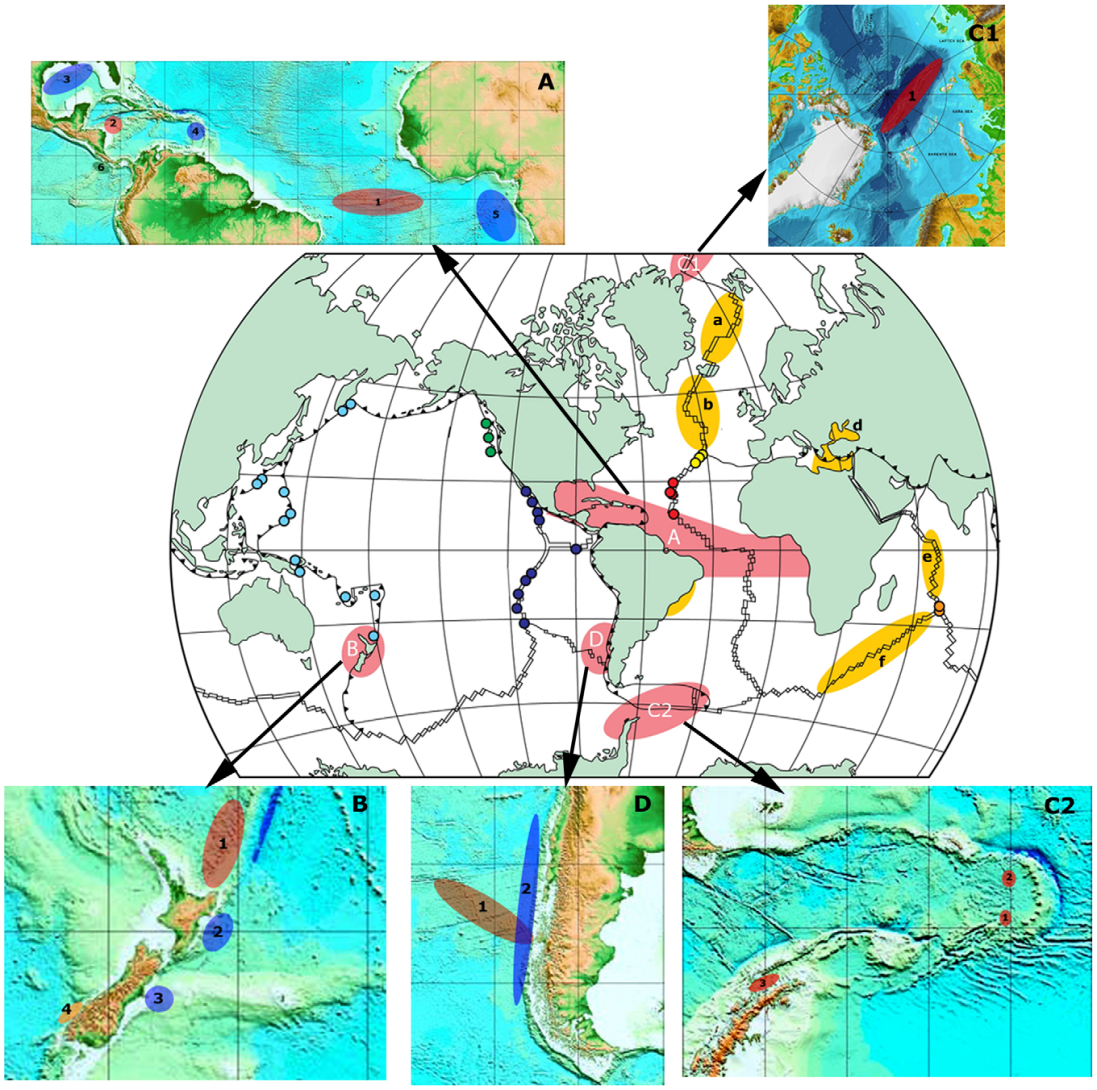
ChEss field program areas. Central map: global map showing the mid-ocean ridge and the known hydrothermal vent provinces (colour dots) known in 2002 (map modified from Van Dover et al., 2002). Highlighted regions indicate the ChEss field program areas, with priority areas (pink) and secondary areas (yellow). Priority areas: A) Atlantic Equatorial Belt; B) New Zealand region, C1) Arctic Ocean, C2) Antarctic region; D) South East Pacific/Chile Triple-Junction; Secondary areas: (a) MAR north of Iceland; (b) MAR north of Azores; (c) Brazilian margin; (d) Eastern Mediterranean; (f) SW Indian Ridge; (g) Central Indian Ridge. Arrows point to detailed maps of each priority field program area. A) AEB: (1) Equatorial MAR and fracture zones; (2) Mid-Cayman Rise; (3) Gulf of Mexico; (4) Barbados Accretionary Prism; (5) NW African margin; (6), Costa Rica margin. B) New Zealand (1) Kermadec Arc; (2) Hikurangi margin; (3) Otago margin; (4) South Island fjords. C1) Arctic basin: (1) Gakkel Ridge. C2) East Scotia Ridge, Southern Ocean: (1) E9 area; (2) E2 area; (3) Bransfield Strait. D) South East Pacific/Chile Triple Junction: (1) East Chile Rise; (2) Chile margin.

While recognising that the ChEss project would need to be largely exploratory in nature, we established that, within the Census decade, the prioritisation of targets for our internationally-coordinated efforts would need to be very selective and only focus upon those most remote or extensive areas of the deep ocean that no single nation could investigate alone. Consequently, while a number of other target areas remain of great scientific interest, only 4 specific areas were targeted by the ChEss project as a whole: A) the Atlantic Equatorial Belt; B) the chemosynthetic ecosystems close to New Zealand; C) the polar oceans of the Arctic (C1) and Antarctic (C2) and D) the Chile Triple Junction (Figure 1). In the Atlantic Equatorial Belt, our goals were two-fold: first, to investigate phylogeographic relationships between vent and seep communities that were already known within the study area and, second, to investigate connectivity and better understand the extent to which geologic factors (e.g. the equatorial fracture zones that offset the northern and southern MAR by >1000 km) and oceanographic processes (e.g. the pathway for North Atlantic Deep Water that flows through these fracture zones) might act as conduits for, or barriers to, gene-flow. In New Zealand and at the Chile Triple Junction, by contrast, our primary goal was to investigate the environmental factors that control the diversity and distribution of the fauna found in different chemosynthetically-driven ecosystems located in close geographic proximity. Finally, in our Polar Region studies, our primary goal was to investigate the potential for isolated genetic evolution within an interconnected global ocean.

## Results and Discussion

### Accomplishments of the ChEss Project (2002–2010): filling the gaps in the global biogeographical puzzle

**Figure 2:**
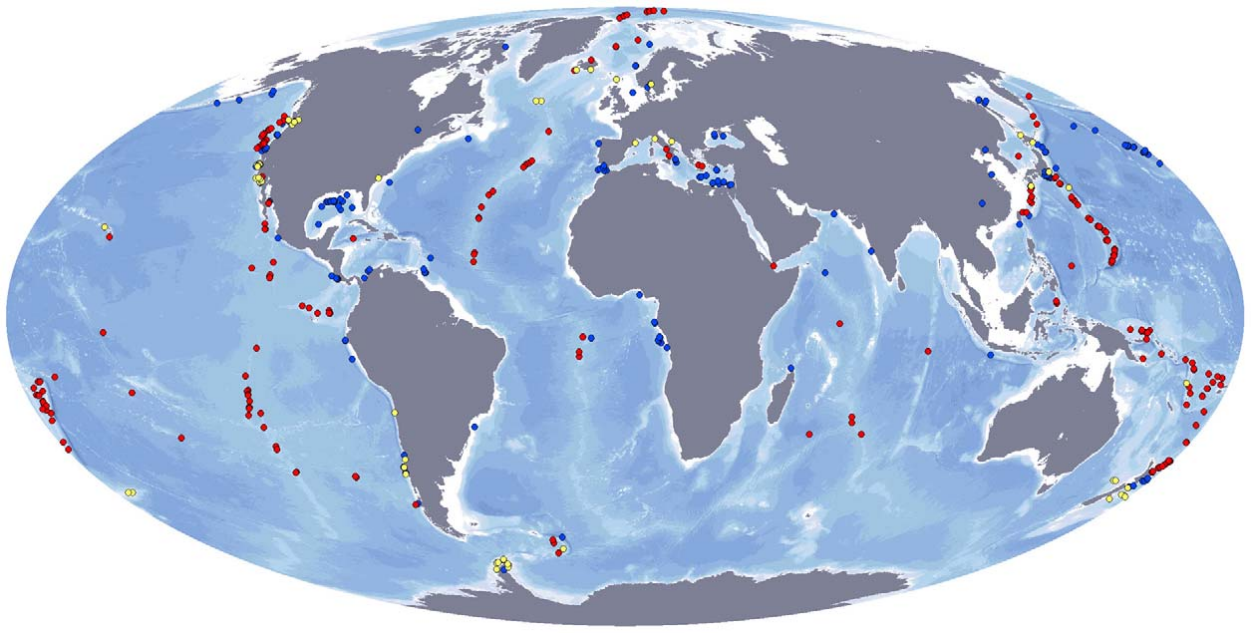
Global distribution of hydrothermal vent (red), cold seep (blue) and whale fall (yellow) sites that have been studied with respect to their fauna. Created by M Baker and D Cuvelier, using site location data gathered from published literature or provided by researchers.

Discovery of deep-water chemosynthetically-driven communities remains recent in the history of ocean science – itself a relatively young discipline. For example, the span of the ChEss project (2002–2010) represents ∼25% of the entire history of chemosynthetic ecosystem research and a further 180 species previously unknown to science have been described during this time – i.e. the rapid rates of discovery established during the last quarter of the 20^th^ Century have continued with no drop in rate throughout the first decade of the 21^st^ Century – the Census of Marine Life decade. The exploration conducted in the four ChEss key areas (Atlantic Equatorial Belt, New Zealand region, Polar regions and South Pacific off Chile) led to a new map of known vent, seep and whale fall sites (Figure 2) and provided the necessary geological framework from which to develop biological studies of faunal communities and their relationships with the environment and other ecosystems. Below, we present the major discoveries obtained in each of the key ChEss areas and discuss the results in relation to global biogeographic patterns of deep-water chemosynthetic ecosystems.

#### The Atlantic Equatorial Belt

The Atlantic Equatorial Belt (AEB) region extends on a longitudinal gradient from the Costa Rica margin in the Pacific Ocean to the continental margin off West Africa, encompassing a very large region around the Equator, from 15°S to 30°N. The key sites include the Costa Rica cold seeps, the Gulf of Mexico cold seeps, the ultra-slow spreading Mid Cayman Rise, the Barbados Accretionary Prism, hydrothermal vents on the Mid-Atlantic Ridge (MAR) north and south of the Equatorial Romanche and Chain Fracture Zones and cold seeps on the continental margin of west Africa (Angola Basin) (Figure 1). The Costa Rica margin, although not in the Atlantic Ocean, was included in this study to investigate the connection pathways between the two oceans prior to the closure of the Isthmus of Panama. The AEB region is characterized by longitudinal topographic barriers such as the Mid-Atlantic Ridge and the Panama Isthmus, and latitudinal barriers such as the Romanche and Chain fracture zones on the MAR. Complex hydrographic features, such as the Northeast Atlantic deep-water currents and equatorial deep jets through fracture zones, are also important environmental factors that may have an effect on dispersal capabilities between chemosynthetic communities in this region.

Our principal interests in the AEB region concerned studying connectivity among different chemosynthetic ecosystems over different length scales and when confronted with different potential oceanographic and geologic conduits and barriers to gene flow. ChEss scientists addressed questions of how fauna might be related across the breadth of the Atlantic Ocean between cold seep sites on the margin of West Africa and those present in the Americas – along the Atlantic margin, the Barbados Accretionary Prism, the Gulf of Mexico and, in the extreme, along the Pacific margin of Costa Rica which would also have been inter-connected with the Atlantic fauna prior to the closure of the Isthmus of Panama. Would oceanographic flow through the deep transform faults of the Chain and Romanche Fracture Zones [27] enhance gene-flow across the Atlantic at these latitudes? Conversely, would these same major fracture zones that offset the northern and southern sections of the Mid-Atlantic Ridge by ∼1000 km act as a significant barrier to gene flow between adjacent vent-sites along the global ridge-crest in this region? Lastly, what might the impact have been of the closure of the Isthmus of Panama at ∼5 Ma [28]? Would hydrothermal vents along the ultra-slow and extremely deep Mid Cayman Rise host fauna that more closely resembled cold-seep fauna from elsewhere in the Gulf of Mexico/Caribbean region, or vent-fauna from either the Mid-Atlantic Ridge or (dating from prior to closure of the Isthmus of Panama) the Galapagos Spreading Center or East Pacific Rise?

#### AEB Pacific-Atlantic connection

The present mid-ocean ridge pathway between the Atlantic and Pacific Oceans runs via the Indian Ocean and Pacific-Antarctic ridge-systems (Figure 1). Prior to the closure of the Isthmus of Panama, ∼5 Ma, a major, low-latitude, deep-ocean gateway existed between these major oceans, albeit with no evident ridge connection [28]. In a generic similarity analysis of cold seeps across this geologically modern Central America land-bridge, the Gulf of Mexico and Florida Escarpment fauna clustered with the Oregon Margin seep communities [29]. Also, the only seep-endemic vestimentiferan tubeworm, *Escarpia* sp., is known both from the Gulf of Mexico and the California slope [30]–[31]. Thus, the limited data available at the onset of the ChEss project suggested a dispersal pathway for seep species between the Pacific and Atlantic via the (now-closed) Panama Isthmus [29]. The Costa Rica margin on the Pacific Ocean side was a key target area within the ChEss-AEB field programme, to address issues of Pacific-Atlantic connectivity. Recent exploration and investigation of the Costa Rica margin revealed a new type of habitat at Jaco Scarp (1792 m), which scientists have named a hydrothermal seep, where methane seepage and diffuse hydrothermal flow are found together [32]. High faunal biomass at the site is comprised of large bushes of *Lamellibrachia* tubeworms, bathymodiolin mussels, *Lepetodrilus* limpets, large vesicomyid clams and galatheid crabs. The fauna exhibits both seep and vent affinities, suggesting the potential for hybrid habitats to provide important connections between different reducing systems [32]. It was also at the Costa Rica hydrothermal seeps that a second species of the Yeti crab *Kiwa* was discovered. As with the vent species *Kiwa hirsuta*, this new species of *Kiwa* farms epibiotic bacteria by waving its bacteria-covered arms in methane seep fluid and then scrapes bacteria off its setae and into its mouth [33].

#### Deepest vents on a Caribbean ultra-slow spreading ridge

**Figure 3:**
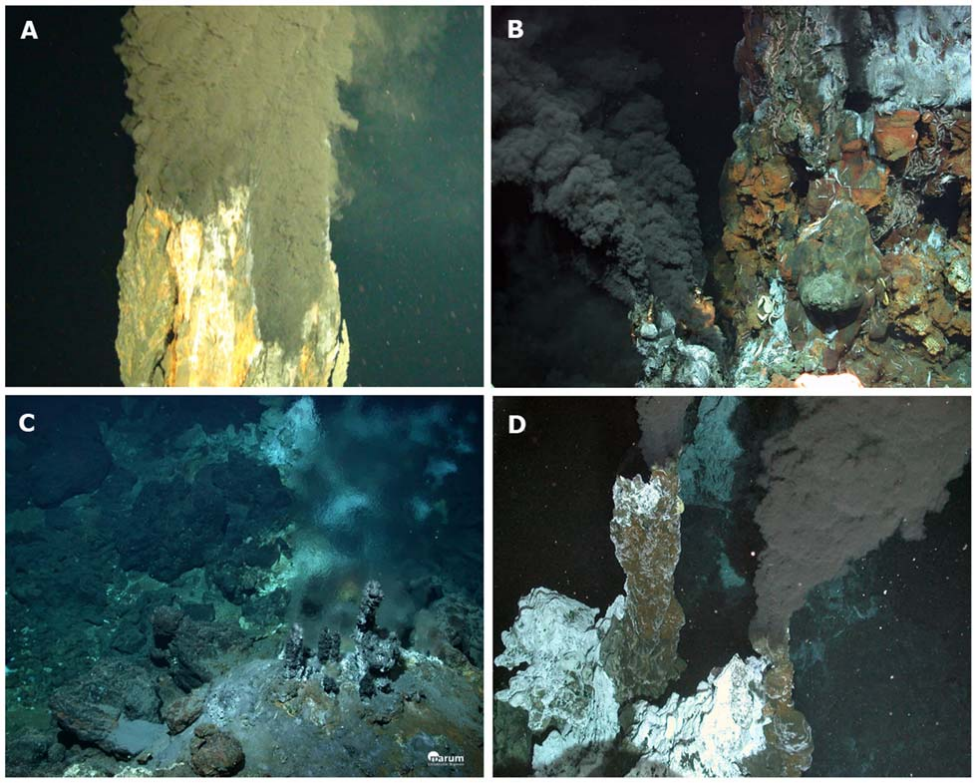
Photos showing some of the newly discovered deep-water chemosynthetic habitats found during ChEss. A) The Beebe Vent, Piccard Hydrothermal Field on the Mid-Cayman Rise (4960 m depth), image taken by HyBIS during Voyage 44 of the RRS James Cook, April 2010 (© Jon Copley, University of Southampton/NOCS) (Connelly et al,. 2011); B, Turtle Pits hydrothermal vent, first vents discovered on the South MAR, 2005 (© ROV Quest, Marum, Bremen); C, Bubbylon hydrothermal vents on the North MAR discovered near the Azores in October 2010 (© ROV Quest, Marum, Bremen); D, Black smokers on the East Scotia Ridge south of the Polar Front discovered in January 2009 (©Paul Tyler and Jon Copley, ChEsSo Consortium, University of Southampton, UK, ROV Isis).

The presence of hydrothermal activity on ultraslow spreading ridges (<2 cm.year^−1^) was only recognized at the end of the 1990s [34]–[37]. Slow and ultraslow ridges, however, represent 50% of the global ridge system [38] and dominate the Arctic, Atlantic and southwest Indian oceans [39], comprising a great diversity of venting styles [40]. The Mid-Cayman Rise is a deep (ca. 5000 m depth), geographically and tectonically isolated ultra-slow spreading ridge located in the western Caribbean Sea. It is located along the path of the past deep-water connection that existed between the Pacific and Atlantic oceans before the closure of the Panama Isthmus, making it an essential study site to understand current patterns of vent biogeography. To address the potential dispersal pathway of vent species between the East Pacific Rise-Galapagos Rift system and the Mid-Atlantic Ridge, the Mid-Cayman Rise was chosen as a key exploration and investigation area within the ChEss project. To that end, a 2009 cruise provided the first evidence for hydrothermal venting at three sites on the Cayman Rise, each indicative of different types of water-rock interaction and including the deepest known vents on Earth [41]. A follow-on cruise, the following year, provided for the first visual observations of any of these hydrothermal vents and their associated fauna [42] Figure 3A).

#### Mid-Atlantic Ridge fracture zones: latitudinal barriers to dispersal?

The Romanche and Chain Fracture Zones, which cross the equatorial MAR, are old (60 Ma) and represent significant topographic features (4 km high–935 km ridge offset) that prominently affect both the linearity of the ridge system and large-scale ocean circulation in this region. Van Dover [43] suggested that geologic features such as the Romanche and Chain Fracture Zones could act as significant topographic barriers, limiting the dispersal of vent fauna along-axis between the southern and northern MAR. The potential for dispersal along any ridge-axis will depend upon the biological and ecological characteristics of the larvae, as well as on the physical parameters (hydrography and bathymetry) created by the presence of, in this case, the equatorial fracture zones. To address these connectivity issues within the ChEss project, the first step involved exploring for hydrothermal vents immediately south of the Chain and Romanche Fracture Zones, i.e. just south of the equator. In 2005, the first hydrothermal vents on the MAR south of the equator (3–7
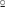
S) were found, with 3 sites identified [44]. High temperature venting was measured at the Turtle Pits and Red Lion vent sites, with fluids at Turtle Pits reaching 407°C at 3000 m depth - conditions close to the critical point for seawater and also the hottest mid-ocean ridge vent-temperatures reported to date [44]–[46], (Figure 3B). High temperature venting was not observed at the adjacent Wide Awake field, but extensive fresh pillow lavas were photographed in this area, including lobes that had flowed over and engulfed colonies of diffuse-flow hosted *Bathymodiolus* mussles, confirming the presence of recent volcanic activity [44]. While subsequent cruises using ROVs investigated the faunal communities of these vent sites, between 2005 and 2009, detailed taxonomic descriptions of those communities are still not available. However, from visual observations at these vent sites, immediately south of the Equatorial Fracture Zones, it would appear that there are very close similarities between the fauna observed here and those reported previously from further north along the Mid-Atlantic Ridge [44]. Some of the dominant fauna include the mussel *Bathymodiolus*, the alvinocarid shrimp *Rimicaris* cf. *exoculata* which does not form dense swarms as in the Northern MAR [46], [47] and the vesicomyid clam *Abyssogena southwardae* amongst others [46], [48], [49]. A recent molecular study on *Rimicaris exoculata* from 5 hydrothermal fields between 36°N and 4°S show that the populations of this shrimp have a very recent common history, with a current large effective population size and/or high dispersal capacity [43], [44], [50]. Although the taxonomic and molecular studies on *Bathymodiolus* and *Abyssogena* and their symbionts are still ongoing, initial results indicate that the equatorial fracture zones on the MAR do not represent a significant barrier to faunal dispersal along the ridge axis [49].

#### North Atlantic Deep Water: conduits for dispersal across basin?

The AEB area of the ChEss project comprises several cold seep sites at either side of the Atlantic, including the Gulf of Mexico seeps, the Barbados accretionary prism, the Blake ridge diapir and the northwestern Congo, Gabon and Nigeria margins in northwest Africa (Figure 1). The Gulf of Mexico cold seeps represent a broad area of seepage resulting from salt tectonics that have been extensively studied between 400 and 3300 m depth [51], [52]. These seeps are dominated by 3 major groups of symbiotic fauna with chemosynthetic methanotrophic bacteria: tubeworms, bathymodiolid mussels and vesicomyid clams. The Barbados accretionary prism results from subduction of the Atlantic plate beneath the Caribbean domain [53]. The major groups in the seep community comprise mussels, vesicomyid clams and vestimentiferan tubeworms, but also include a large number of sponges, gorgonians and corals [53]. Mud volcanoes and their associated seep fauna - including sponges with methanotroph endosymbionts - also occur in the Barbados Trench at 5000 m depth [54]. The Blake Ridge diapir is associated with methane gas hydrate and dominated by extensive mussel beds of large *Bathymodiolus heckerae* that can reach over 36 cm in length and smaller vesicomyid clams of the species *Vesicomya* cf *venusta*) [25]. Recently, cold-seep communities have been investigated in the African margin, on pockmarks in the Gulf of Guinea, Congo, Gabon and Nigeria margins, dominated by mytilid mussels, vesicomyid clams and/or siboglinid tubeworms, [55], [56]. A study comparing 72 taxa from this AEB site at the species level revealed that 9 species or species-complex are amphi-Atlantic [57]. That study suggests that depth is the main factor affecting megafauna community structure, with depth segregation observed between 1000 and 2000 m. Conversely, geographic distance does not explain the patterns observed and the highest community similarity was found between the fauna from the Florida Escarpment seeps and the fauna from the Congo margin, at either side of the Atlantic Ocean [57]. Furthermore, the MAR vent sites did not appear to act as stepping stones for dispersal across the basin. The factors driving connectivity of seep species across the Atlantic are still unclear. Olu-Le Roy and co-authors [57] suggest different hypotheses that could explain the current species distribution on Equatorial Atlantic seeps. One possibility is that what appear to be shared species are actually cryptic species; further molecular studies are needed to resolve this issue. However, the amphi-Atlantic distribution of some species may also be explained by larval ecology and deep-water currents. Van Dover et al. [25] suggested that the southward flow of North Atlantic Deep Water along the American east coast and its eastward flow along Potential Vorticity contours – a variable measured as the ratio between ocean depth and the Coriolis force [58] – near the equator might explain the distribution of chemosynthetic species in the NW Atlantic. For example, the vesicomyid clam *Calyptogena* aff. *kaikoi* is not only present at the Florida Escarpment seeps and the Barbados Accretionary Prism, but is also found at the Logatchev vent site on the MAR near 15°N [59]. Similarly, the mussel *Bathymodiolus heckerae* is found at both North Carolina and Florida Escarpment seeps, while closely related species are also found at MAR vent-sites [25]. North Atlantic Deep Water flows south along the East coasts of North and South America as far as the Equator before being deflected east, crossing the MAR through conduits created by the Chain and Romanche fracture zones [27]. Deep circulation within these fracture zones is turbulent and may provide an important dispersal pathway for species from west to east across the Atlantic [25].

One of the most recent discoveries on the Mid-Atlantic Ridge occurred in October 2010, some 500 km southwest of the Azores. New vents with metre-high chimneys were discovered here hosting marine life typical of that found on other MAR vents (Figure 3C). This area is outside the AEB region, but the findings have been included here as they were based on technologically exciting developments, a major aspect of the ChEss project, where scientists used a latest-generation multi-beam echo sounder that allows the imaging of the water column above the ocean floor with previously unattained precision [60].

#### New Zealand Region

The oceans around New Zealand were chosen as a specific site of interest because multiple types of chemosynthetic ecosystems can be expected to exist in reasonably close proximity (Figure 1), presenting a useful natural laboratory to investigate the extent to which habitat variability might influence the nature of the fauna found at differing chemosynthetic sites. At the onset of the ChEss project, there were known vent sites to the north of New Zealand along the Kermadec volcanic arc that links to the Lau basin. There was also some indirect evidence of cold seep habitats on the Hikurangi margin east of North Island and off Otago on the South Island via seep-type fauna identified from fisheries by-catch, but no *in situ* images of active cold-seep communities had been obtained, nor had any comprehensive sampling of live cold-seep fauna been undertaken. The fjords of the south-west coast in the South Island are potential target regions for oxygen minimum zones and large organic falls, including sunken wood and kelp. Furthermore, the seas around New Zealand are known as an important whale migration route and whales are particularly abundant in certain areas, providing the likelihood of important seafloor deposits in these locations. Areas provisionally identified as suitable and accessible for whale skeleton investigations included the Kaikoura canyon (east coast South Island) and the Cook Strait canyons (between the North and South islands). Exploring for and investigating deep-water chemosynthetic ecosystems and their fauna in this region would fill a significant gap in our understanding of the global biogeography of chemosynthetically-driven species.

Perhaps the most exciting result from the ChEss project in this region was the discovery of 10 new cold seep sites along the Hikurangi margin, including one extremely large site, the Builder's Pencil site, that covers 135,000 m^2^ [61]. The fauna is mainly composed of vesicomyid clams, bathymodiolin mussels, siboglinid tubeworms, Ampharetidae, Dorvilleidae and Spionidae polychaetes, as well as an encrusting sponge of the genus *Pseudosuberites*. Although many of these groups are also common in other cold-seep areas, there were significant differences at the species level, with most species being new to science or endemic to New Zealand seeps, suggesting that the New Zealand cold-seep communities could represent a new biogeographic region [61]. Support for this argument is found in other New Zealand chemosynthetic ecosystems, such as the Kermadec Arc hydrothermal vents and sunken wood and whale falls collected around New Zealand, where species compositions also show significant differences from those of other known regions, A. Rowden, *pers. comm.*]. A key objective of the ChEss programme was to address ecological and evolutionary connectivity amongst different chemosynthetic habitats (i.e. vents, seeps, whale falls and sunken wood) when located in close proximity to one another. Although sampling and species identification is still preliminary in the New Zealand region, initial results show that 4 species are found in all 4 ecosystem types present, representing a 20% overlap in the fauna described to species level [61]. The same study also suggests an ecological connectivity with Antarctic chemosynthetic communities through the Circumpolar current.

#### Polar regions – Arctic

Evidence for venting at both poles was already well established at the outset of the Census decade [36], [62], [63], but no visual evidence of the vents and their potential fauna was available. Recognising the difficulty of conducting deep seafloor research at such high latitudes and the importance of describing their faunal communities to understand global biogeography patterns, ChEss selected these areas as being of particular importance for coordinated chemosynthetic biological research in these extreme but contrasting environments.

Work conducted during this first decade of the 21^st^ Century has revealed evidence for abundant hydrothermal activity on the Gakkel Ridge in the Arctic Ocean [36]. The Gakkel Ridge is an ultra-slow spreading ridge stretching 1800 km across the eastern Arctic basin (Figure 1) beneath permanent ice cover. An important feature of the Gakkel Ridge is that it is located within the bathymetrically isolated Arctic Basin, thereby posing very interesting biogeographical questions. While the timescale for global thermohaline circulation is only ∼2000 years (hence, short compared to the timescale for biological evolution), the Arctic represents an isolated deep ocean basin, surrounded almost entirely by continental land-masses with only relatively shallow sills through which deep-ocean waters can exchange with the Pacific and Atlantic. The deep Arctic water is isolated from deep-water in the Atlantic by sills between Greenland and Iceland and between Iceland and Norway, and from the deep Pacific by the shallow Bering Strait. This has important implications for the evolution and ecology of the deep-water Arctic vent fauna. The Atlantic and Pacific were once connected via the open Arctic Ocean, and their vent fauna could have used this pathway to disperse across ocean basins. Identifying which species are found on the Gakkel Ridge and assessing their ecological and phylogenetic relationships with vent species from Pacific and Atlantic vent fauna will allow us to test the hypothesis of a past Arctic Ocean link. Also, exploring the Arctic ridges will enable identification of new species with specific adaptations to unique ecological habitats. Understanding the evolutionary and ecological processes shaping Arctic vent communities is essential to our understanding of the distribution of vent-fauna across ocean basins. A cruise to the Gakkel Ridge in 2007 [64] provided new information via a fiber-optic drift camera system on the volcanic and hydrothermal processes taking place in this ridge [65], [66] and succeeded in locating sites sufficiently well to image bacterial mats and associated shrimp, amphipods and sponges from a drift camera [67]. However, a return to this and other known vent-locations awaits development of new technologies such as Hybrid Underwater Robotic Vehicles that have only recently begun to be pioneered in geologically-relevant (but lower-latitude) settings [41], [68], [69]. These new underwater vehicles, such as Nereus, from the Woods Hole Oceanographic Institution, can swim freely as an autonomous underwater vehicle (AUV) to explore and map large areas of the deep sea and using chemical sensors to prospect for and locate chemosynthetic systems at the seabed. Then, once a specific target has been identified, the vehicle can be brought back aboard ship and reconfigured as a lightly-tethered remotely operated vehicle (ROV) only connected to the ship by a single optical fibre, comparable in diameter to a human hair. This, allows sufficient bandwidth for telemetry of commands and data, up to and including HDTV video transmission, in real time between the vehicle and the operations team while, simultaneously, providing the vehicle with the freedom of movement to range independently, unlike conventional ROVs, over kilometers or even tens of kilometers away from the mother ship [68], [69].

In the Norwegian-Greenland Sea, the Knipovich, Mohns and Kolbeinsey ridges extend from the south of Svalbard to the north of Iceland. The first two of these are ultra-slow spreading ridges, similar to Gakkel while the Kolbeinsey is a slow spreading ridge, more similar to the Mid-Atlantic Ridge that lies immediately to the South of Iceland. These ridge segments are isolated from the Gakkel Ridge by shallow sills connecting to the Arctic Basin, but are even more dramatically isolated from the MAR by Iceland, where the ridge becomes subaereal. Thus, this system offers a further natural laboratory for evolution on an isolated section of ridge. Although evidence for hydrothermal venting on both the Kolbeinsey and Knipovich Ridges pre-dates the start of the ChEss project [70], [71], no information on associated fauna was available until a suite of shallow (500–750 m) sites were investigated on the Mohns Ridge near 71°N [72]. These sites host extensive mats of sulfur-oxidising bacteria, but only one potentially symbiont-bearing multi-cellular taxon – the small gastropod *Rissoa* cf. *griegi* – which is also known from North Atlantic cold seeps and wood falls. At a similarly northerly chemosynthetic setting, on the Haakon Mossby Mud Volcano near 72°N on the Norwegian margin, colonization of active cold seeps is dominated by siboglinid worms [73].

#### Polar regions – Southern Ocean

The Bransfield Strait and East Scotia Ridge are isolated back-arc basins located at the gateway from the Pacific to the Atlantic sector of the Southern Ocean (Figure 1), where evidence of hydrothermal activity was identified in the early 2000s [62], [63], [74]. Three hypotheses were developed to explain the potential faunal colonization of these sites. If colonization of the Bransfield Strait/East Scotia Ridge vents was driven by hydrography, then the faunal composition should be related to that of Pacific vents, with propagules dispersing with the Antarctic Circumpolar Current from the Southern East Pacific Rise and/or Chile Rise. If, on the contrary, the colonization of the Bransfield Strait and East Scotia Ridge vents followed geological controls, then the fauna may have migrated to these sites across the seafloor and more closely resemble those of the southern Mid-Atlantic Ridge. The third alternative is that the fauna of the Bransfield Strait and/or the East Scotia Ridge may have evolved quite independently from both East Pacific Rise and Mid-Atlantic Ridge vent-fauna [62]. Certainly, a dispersal pathway from Pacific to Atlantic via the connection of the Chile Rise, the Antarctic Ridge (Scotia Sea) and the Mid-Atlantic Ridge did exist previously, during the Tertiary [29].

The most important result of ChEss in the Southern Ocean region was the first direct observation and sampling of vent communities on the East Scotia Ridge south of the Polar Front in 2009 [75](. Two vent fields were observed with the SHRIMP towed camera system, hosting both black smokers and diffuse venting (Figure 3D). The preliminary faunal community analyses indicate that the East Scotia Ridge vents represent a new biogeographic province, isolated from other chemosynthetic ecosystems by the Polar Front that forms a major physiological barrier to dispersal [75]. Some connection between the vent communities found on the East Scotia Ridge with those from the Southern East Pacific Rise, Chile Rise and Southern Mid-Atlantic Ridge would be possible through larval dispersal mediated by the Antarctic Circumpolar Current. Further analyses are, however, necessary to provide a robust explanation of connectivity between the different southern latitude ocean basins.

In addition to the Southern Ocean hydrothermal vent sites recently discovered, a new cold-seep habitat was recently described from the Antarctic. Following the collapse of the Larsen B ice shelf at the southeastern margin of the Antarctic Peninsula in 2002, Domack and colleagues [76] provided first videographic evidence of potential chemosynthetic communities in this area. Further video investigations in the framework of the Census of Marine Life CAML (Antarctic) and ChEss projects found shells of the vesicomyid clams of the genus *Calyptogena* sp., but no living animals nor thiotrophic bacterial mats were observed [77], suggesting a declining chemosynthetic ecosystem. Domack et al. [78] have postulated that, while glacial conditions (ice shelf) favour the development of chemoautotrophy on the seafloor, the rapid sediment flux caused by environmental catastrophes such as rapid deglaciation may trigger significant changes in seafloor communities.

#### South Pacific – Chile Triple Junction

The Chile Triple Junction has represented a particularly important locale for the ChEss project because it is the only place on Earth where an actively spreading ridge is currently being subducted beneath a continental margin [79] (Figure 1). The East Chile Rise also represents one of the past geologic pathways between the Pacific and Atlantic Oceans, because it was formerly connected to a complex mid-ocean ridge and subduction zone system that connected across from the southern East Pacific Rise to the Atlantic, between the tip of South America and the Antarctic Peninsula [80]. For the last ≥10 Ma, however, the East Chile Rise has been isolated from the Antarctic ridge system by subduction beneath South America. Nevertheless, a significant component of the Antarctic Circumpolar Current passes over this section of ridge-crest before flowing through the Drake Passage and past the Bransfield Strait where the Antarctic Circumpolar Current diverges, with a significant component continuing east, south of South Georgia and across the East Scotia Ridge. Therefore, identifying and describing the hydrothermal fauna of the Chile Triple Junction and comparing vent species from the southern East Pacific Rise, the Chile Triple Junction, the Bransfield Strait and the East Scotia Ridge would provide for an excellent investigation of the possibility for gene flow of chemosynthetic species across ocean basins around South America. At the onset of the ChEss project, the only hydrothermal information available on the Chile Triple Junction was evidence of metalliferous input to the sediments in this region [81]. Most recently (March 2010), evidence has been obtained for two differing potential hydrothermal sources within 5 km of the Chile Triple Junction, one of which may be sediment hosted [82], and which, again, are poised for future chemosynthetic biological investigation.

Another important aspect of investigating the SE Pacific region off Chile was that every known form of deep-water chemosynthetic ecosystems could be expected to exist in this area: hydrothermal vents, cold seeps, whale falls and oxygen minimum zones. The Peru-Chile margin and subduction zone contains massive methane-hydrate deposits [83] and seep sites venting methane-rich fluids, although these habitats were largely unexplored along the SW Chile slope at the onset of ChEss. Cold-seep communities were first reported from the Chile margin off Concepción by Sellanes et al. [84] and these, when examined in detail, showed important differences from “nearest-neighbour” seep communities both further north along the same continental margin, off Peru, and at comparable latitudes to the west, off New Zealand [61], [85], [86].

At shallower depths, between 200 m and 500 m, the Chile continental margin is intercepted by a well-developed oxygen minimum zone [87]. Where this OMZ impinges on the seafloor, dense mats of sulfur-oxidizing bacteria (*Thioploca*) cover the sediments. Isotopic evidence and the occurrence of some symbiont-bearing species indicate that the nutrition of infaunal assemblages in this zone may rely on chemosynthesis [9]. Furthermore, many species of whales feed in the productive waters of the Peru-Chile margin (including minke, southern right, blue and humpback whales) and migrate through this area, moving between summer and winter feeding and breeding grounds [88]. Similarities among vent, seep, OMZ and whale-fall communities in close proximity at the Chile Triple Junction remain poorly known thus far but remain ripe for novel future investigations and discoveries.

### Beyond the First Census of Marine Life: a forward look for deep-ocean chemosynthetic research

#### Continuing exploration

Because of the relative youth of the field of deep-ocean chemosynthetic research, it is perhaps inevitable that the work remaining to be done at the closure of the ChEss project continues to include a strong exploratory element. More than 150 different hydrothermal vent-fields have been identified along that ∼20% of global mid-ocean ridges that has been investigated thus far, at an average spacing approaching ∼100 km between adjacent sites [89]. Since it is now known that hydrothermal fields can occur in all ocean basins and that this incidence of venting is sustained along even the least volcanically active slow- and ultraslow-spreading mid-ocean ridges [22], [32]–[34] then we can calculate that there should be at least 500 and perhaps as many as one thousand high-temperature hydrothermal fields in total along the global ridge crest. Even by the most conservative estimates, therefore, it is clear that that there should remain more high-temperature hydrothermal vent-fields waiting to be discovered than all those that have been identified since the first discovery on the Galapagos Rift in 1977.

Of course, chemosynthetic fauna do not only colonize high-temperature hydrothermal fields. Typically, the highest concentrations of biomass associated with any hydrothermal field are found hosted in low-temperature diffuse-flow settings and it is now recognized that low-temperature systems like the Lost City hydrothermal field can occur, away from the presence of any high-temperature venting, in a geologic setting that recurs along the slower-spreading ∼30,000 km of the global ridge crest [38], [90]. To date, however, we know of only one investigation that has set out to explore systematically for both low-temperature as well as high-temperature hydrothermal venting along any section of the global ridge crest [37], [42] and that survey located evidence for 2 high-temperature fields and 2 low-temperature systems along ∼100 km of ultra-slow spreading ridge. From that preliminary data set, and knowing that the geologic setting of such sites is restricted to that half of the global ridge crest that is slow or ultra-slow spreading, then we can extrapolate to the global scale and estimate that there may be a further ∼300–600 low-temperature hydrothermal systems (beyond the three that are currently known) along that section of the global mid ocean ridge system that extends from the Gakkel Ridge in the Arctic, along the entire length of the Mid Atlantic Ridge and continuing along the SW Indian Ridge as far as the Rodriguez Triple Junction. Given the paucity of data upon which such an extrapolation is based, however, the accuracy of this prediction must be recognized to be unknown, if not unknowable.

Along ocean margins, cold-seep ecosystems have been located along both active and passive geologic margins (i.e. those with and without active subduction zones, respectively) from the South Pacific to the Norwegian Greenland Sea as well as at multiple locations in between (Figure 2). Within our Atlantic Equatorial Belt alone, cold seep ecosystems are known along the margins of Costa Rica, the Gulf of Mexico, the Barbados Accretionary Prism and the Gulf of Guinea (Figure 1A). As with the earliest stages of mid-ocean ridge hydrothermal research, however, those sites that are currently known have primarily been located through serendipity rather than systematic exploration and the geographic distributions of sites that have been located are heavily aliased toward regions of greatest human activity (e.g. hydrocarbon exploration in the Gulf of Mexico, USA) rather than necessarily reflecting their global distribution and abundance. As with the case for low-temperature hydrothermal venting, however, at least some preliminary estimates can be made on what the relative abundance of cold-seep ecosystems may be, worldwide. By definition, every mid-ocean ridge that originates through rifting of a continental land-mass (e.g. the modern day East African rift-valley, Red Sea and Bransfield Strait) is bordered by two conjugate margins. Exceptions can occur – e.g. in the case of the back-arc systems of the Western Pacific Ocean and the East Scotia Ridge - but if this assumption holds for the majority of the mid-ocean ridge crest (say 50,000 km) then we can calculate that there must be ∼100,000 km of ocean margin that can potentially host methane-rich “cold seep” fluid flow. If we then assume that cold-seep fluid flow recurs, on average, every 100 km along all ocean margins (in the absence of sufficient data to constrain a more intelligent estimate) then this would suggest that a total of ∼1000 cold-seep sites may exist, world-wide. Considering that >100 such sites are already known but that systematic methods for exploration for cold seeps only began to be pioneered toward the very end of the Census of Marine Life decade [82], [91] we do not consider this estimate unreasonable.

While much exploration remains to be done, however, the achievements of the ChEss project have not been without significant reward. During the 8 years of the project we have not only documented some 180 species previously unknown to science (see earlier), but also extended the limits to which “extreme” life can exist with the discovery of both the hottest known vents (407°C, [45]) and the deepest (∼5000 m, [41], [42],). Whilst our ChEss-coordinated activities have also helped to locate the first vents in the SW Indian, S Atlantic and SE Pacific oceans, as well as in the Southern Ocean itself, a number of questions remain unanswered:

- In the global ocean how many symbiotic, chemosynthetic species exist?
- What are their distributions and how are those distributions controlled?
- How do fauna from chemosynthetic habitats reproduce, disperse and colonize new sites?
- What are the evolutionary histories and phylogeographic relationships of species from different chemosynthetic habitats?

**Figure 4:**
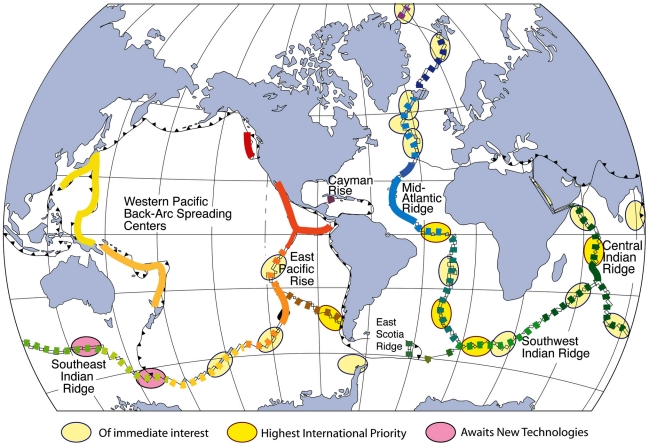
Annotated map (after Van Dover, 2010) of the global ridge crest, illustrating both a model of the global biogeographic differentiation of invertebrate species associated with hydrothermal vents and regions recently identified by the international community as being of continuing importance for future ridge-crest exploration. Ellipses show three categories of importance assigned to twenty three separate locations distributed along the ridge axis (see Table 1); different coloured lines along-axis represent regions that share many of the same species (see Van Dover, 2010).

A number of the vent-discoveries achieved during the ChEss project relied heavily upon the advent of the emergent technology - notably deep-ocean autonomous underwater vehicles (AUVs) (e.g. [92], [93]). This trend was particularly well represented at a workshop convened by InterRidge's *Long Range Exploration* working group held in June 2010 in the UK: (http://www.interridge.org/en/WG/Exploration/workshop2010). At that meeting, a series of targets for future ridge-crest exploration were identified. There, of 24 sections of mid-ocean ridge considered for future exploration, 23 represented areas where either no hydrothermal investigations have yet been undertaken (e.g. southern Mid-Atlantic Ridge south of 15°S; Oblique SWIR, 0–10°E) or where hydrothermal activity is known to exist based on hydrothermal plume studies, but no detailed investigations of the underlying seafloor have yet been undertaken sufficient to provide even a cursory knowledge of what chemosynthetic fauna might be present (e.g. Gakkel Ridge, Pacific-Antarctic Ridge). Consistent with the relative youth of the field of deep-ocean chemosynthetic ecosystem research and the large gaps in knowledge that persist, the need for continuing exploration remains strong and all 23 of these regions were identified as of potential significance to improving our understanding of what controls the biogeography and biodiversity of chemosynthetic ecosystems worldwide. Within the multi-disciplinary framework of InterRidge, however, these 23 locales were then ranked according to their potential significance to researchers working in 4 further sub-disciplinary criteria to help establish priorities for the next decade of internationally coordinated mid-ocean ridge research (Table 1). Researchers representing each of 5 sub-disciplines (geology and geophysics, hydrothermal geochemistry, physical oceanography, biogeography and astrobiology) were tasked with nominating their 5 highest priorities for future exploration. Within the sub-field of vent biogeography, 3 of the 5 preferred candidate regions were found to match well to the interested of the broader InterRidge Community: the Equatorial Fracture Zones of the Mid-Atlantic Ridge, the southernmost Mid-Atlantic Ridge and the easternmost SW Indian Ridge. Together, these three regions represent a large gap in our knowledge that is situated at a juncture between (i) the MAR biogeographic province to the north that appears to extend across the Equator to at least 10°S, (ii) the Southern Ocean province identified most recently from our discoveries along the East Scotia Ridge and (iii) an Indo-Pacific province that appears to extend from the Rodriguez Triple Junction all the way to the SW Pacific (Figure 4). The other two major priorities for future exploration for vent biogeography (as opposed to biologic studies of sites that are already known to exist – e.g. Gakkel Ridge, Mid-Cayman Rise) were on the Central Indian Ridge, north of the Rodriguez Triple Junction and along the East Chile Rise, close to and away from the Chile Triple Junction. As described earlier, the Chile Triple Junction remains of interest because of the recently-demonstrated close proximity of deep-ocean hydrothermal vent and cold-seep habitats in close (<50 km) geographic proximity [82] While we were only able to complete the most cursory preliminary exploration of this region within the lifetime of the ChEss project, that was nevertheless sufficient to establish this region as an ideal natural laboratory for future chemosynthetic ecosystem research. The northernmost Central Indian Ridge is of interest because this section of the NW Indian Ocean represents a modern-day “cul de sac” for deep ocean circulation (hence, potentially for gene-flow as well) that may, however, also preserve evidence for past genetic links to the North Atlantic via hydrothermal fields in the Tethys Ocean, prior to closure during the tectonic collision of Africa and Eurasia [94]. While the Australian-Antarctic Discordance and Macquarie Triple Junction in the southernmost Pacific Ocean were also identified of extreme potential importance, investigations of those areas were considered premature as we continue to await delivery of new next-generation long-range autonomous underwater vehicles suitable for sustained operations at high latitudes in these areas [95]. In a parallel development, very recent work has shown the value of using in situ sensing from deep ocean AUVs, together with geophysical remote sensing, to search for, systematically, and locate sites of active fluid flow – and associated chemosynthetic ecosystems – along both active and passive ocean margins [82], [91]. In the decade ahead, therefore, it is conceivable that systematic cold-seep exploration along ocean margins could become as established as AUV-based hydrothermal exploration has become during the lifetime of the ChEss project.

**Table 1:** Geographic areas of interest for the next decade of internationally coordinated mid-ocean ridge research.

	Geology & Geophysics	Hydrothermal Geochemistry	Physical Oceanography	Biogeography	Astrobiology	Total
01. Gakkel Ridge			1	√	1	2
02. Knipovich Ridge	√	√	1	√		1
03. Reykjanes Ridge		√		√		0
04. Charlie Gibbs FZ	√	√	√	√	1	1
05. MAR North Azores				√		0
06. Equatorial Fracture Zones	1	1	1	1	1	5
07. South MAR 15–35°S	1	1	1	√		3
08. South MAR 35–55°S	√	√	√	1		1
09. Oblique SWIR (0–10°E)		√	√	1		1
10. SWIR (20–30°E)	1	√	√	√		1
11. SWIR (60°E)		1		√		1
12. Central Indian Ridge	1	√	√	1		2
13. Carlsberg Ridge		√	1	√		1
14. Red Sea		√	√	√	1	1
15. Andaman Sea	1	√		√		1
16. SEIR (80–130°E)			√	√		0
17. AustAntDiscord	1	1		√		2
18. Macquarie TJ	√	√	√	√	1	1
19. South Marianas Trough	√	√				0
20. PacAntRidge (N Polar Front)				√		0
21. PacAntRidge (S Polar Front)			√	√		0
22. South EPR (14°S and below)		√		√		0
23. Bransfield Strait	√	√	√	√		0
24. East Chile Rise	√	1		1		2

Areas of interest to any of the 7 sub-disciplines represented among the workshop participants (InterRidge Long Range Exploration working group, 2010 workshop, NOCS, UK) were first identified by ticks. Each disciplinary group then assigned highest prioritization to 5 key areas (marked as 1). Total indicates the number of high priority hits for each geographic area.

#### Anthropogenic impacts

What have become increasingly apparent during the lifetime of the ChEss project are the increasing anthropogenic threats, both potential and real, to chemosynthetic ecosystems in the deep sea. Human activities such as deep-water fisheries, mining, hydrocarbon exploitation and littering are moving into deep waters faster than we can begin to understand the abundance, diversity, distribution and functioning of the fauna in unperturbed ecosystems [96]. In the case of fisheries, evidence that trawling has impacted cold-seep sites has been documented at 500 m depth along the California margin [97] and on the Chile margin where the commercially important Patagonian toothfish appears to be intimately associated with cold seeps [86]. Most recently, the excitement of the discovery of new cold-seep sites off New Zealand was tempered by the recognition that these sites had already been subjected to the impacts of bottom trawling - evident from visible accumulations of vesicomyid shell debris, coral rubble, lost trawl gear and trawl marks, through seep-adjacent sediments [61], [98].

With the depletion of land-based and shallow-water resources, the hydrocarbon industry has also begun to conduct oil and gas exploitation from beneath the deep ocean floor. Large subsurface hydrocarbon reservoirs often give rise to cold seeps along ocean margins such as off West Africa and the Gulf of Mexico. Collaborations between the relevant regulatory authorities and ocean scientists have often proceeded well (e.g. [51], [99]) and exploration for hydrocarbons can often help lead to scientific discoveries of new seep sites. It is anticipated that this healthy symbiosis in research and environmental assessment will continue into the future, for example through initiatives led by the Total Foundation in France and the new Bureau of Ocean Energy Management, Regulation and Enforcement in the USA. However, following the recent oil spill from the Deep Water Horizon platform in the Gulf of Mexico, the largest accidental spill in history, it has been estimated that the majority of the oil released may have been transported away from the site via deep-water plumes that it has been identified dispersed south and west away from the Macondo well-head, never reaching the ocean surface [100]. Furthermore, recent ROV-based visual observations that deep-water coral close to the accident appeared to be impacted by the spill (C. Fisher, pers. obs.; press release and photos at http://www.science.psu.edu/news-and-events/2010-news/Fisher11-2010) demonstrate that anthropogenic impacts resulting from deep-water hydrocarbon extraction need not be restricted to upper ocean and coastal ecosystems [100]–[102].

Perhaps the gravest long-term threat to chemosynthetic ecosystems in particular, however, will come from the emerging marine mining industry. The seafloor massive sulfide deposits formed around submarine hydrothermal vents have the potential to be commercially viable for the extraction of important base metals, notably copper, zinc, silver and gold [103]. While this industry remains in its infancy, the prospect appears very real that commercial extraction from the seafloor, beginning within the Exclusive Economic Zones of various SW Pacific island nations, will begin within the coming decade [104], [105]. Like the methods to be used for mineral extraction, the potential impacts upon seafloor chemosynthetic communities remain poorly constrained. Key areas of consideration, however, include: physical damage of the seafloor and perturbation of pathways of fluid flow, extinction of rare species and potentially of isolated populations, smothering of filter feeders from deposition of sedimentary plumes caused by seafloor activities, noise pollution, waste-water disposal, and potential equipment failure and leakage at the seabed. Throughout its lifetime, the ChEss project has been active in coordinating discussions about the way forward in this field, promoting dialogue with interested parties from the wider Earth and Ocean Science community through the InterRidge Seafloor Mineralization Working Group (www.interridge.org/en/node/5797) and with regulators and emerging industrial interests through the International Seabed Authority(ISA, 2004). It is to be hoped that concerned scientists will continue to be engaged at the international scale in developing protocols and safeguards that can be adopted as part of an international standard for regulating deepwater mining in the future – especially with the recent demonstration that even “extinct” hydrothermal-vent systems may host endemic chemosynthetic fauna [106].

That the scientific community stands ready to meet these challenges and engage constructively with industry is already proved. The adoption of a voluntary code of conduct for responsible research at and near hydrothermal vents [107] has already proved a valuable precursor to the subsequent drafting of The Code for Environmental Management of Marine Mining that is currently under development by the International Marine Minerals Society (http://www.immsoc.org/IMMS_code.htm). In a parallel development, the OSPAR convention (Oslo-Paris Convention for protecting and conserving the North-East Atlantic and its resources; http://www.ospar.org/) has also proceeded with the design of their own Code of Conduct for Research on Hydrothermal Vents [108]. Most recently, a community workshop was held in 2010 aimed at developing a protocol for the *Design of Marine Protected Areas for Chemosynthetic Ecosystems Potentially Threatened by Human Activities in the Deep Sea* [109] that builds upon the protocols previously established for the establishment of preservation reference areas for nodule mining in the Pacific Ocean Clarion-Clipperton Zone [110].

#### A synthesis-based study of Deep Ocean Realms: The South Pacific Ocean

For the past ∼30 years, investigations into the diversity, ecology and evolution of different deep-ocean ecosystems (abyssal, seamount, hydrothermal vent, cold seep, organic fall and continental margin) have generally been conducted in relative isolation. Nowhere has this been more apparent than in the Census of Marine Life programme, which established a series of separate field projects to study each environment. Only toward the end of the Census decade did a synthesis of the efforts from those groups come together as the SYNDEEP initiative, which recognised that our future understanding of both (i) the fundamental processes structuring deep-ocean life and (ii) marine management and conservation, are critically dependent upon how well we can integrate our knowledge across these diverse ecosystems and how well we can impart this knowledge to the wider non-scientific community [96] [111]. As just one example, it is only in the past decade that genetic studies of different chemosynthetic ecosystems have revealed that some fauna inhabiting vents, seeps, whale falls and sunken wood share closely inter-twined evolutionary histories [5], [112]–[117]. While individual Census programmes have synthesised data on their main focus (to determine community compositions and structures and the dynamics of processes active in each habitat type), an important strategy that is now being developed, beyond the Census, is the emerging INDEEP programme (www.indeep-project.org) that will train a new generation of researchers equipped to understand better the biodiversity and functioning of deep-sea ecosystems, as well as the connectivity between habitat types. An important goal of INDEEP, as a direct consequence, will be to help meet a pressing need for improved management and conservation of these vulnerable ecosystems. Going forward, it will be critical to (i) evaluate species distributions and differences in community composition and structure in areas that overlap in depth and habitat type; (ii) assess heterogeneous nutrient sources and diverse inputs; and (iii) reveal the genetic relationships among species and populations across diverse deep-ocean habitats. Only then can we expect to understand the formative ecological and evolutionary processes that have shaped current patterns of biodiversity and ecosystem function and serve to sustain deep-sea biodiversity, as well as fisheries-based goods and services in, the face of increasing anthropogenic impacts.

One high-priority area in which we recommend that we should seek to address both long-standing and recently-developed hypotheses is the South Pacific Ocean. This deep-ocean basin represents the largest contiguous ecosystem for life on our planet and contains the full range of known seafloor habitats: vast abyssal plains underlying the huge central oligotrophic gyre, eutrophic hotspots underlying equatorial and Antarctic upwelling zones and active and inactive seamount chains that extend from ocean margins into the ocean interior where they intersect mid-ocean ridges. Furthermore, the South Pacific Ocean also contains the world's most abundant hydrothermal vents, the world's largest seep sites, oxygen minimum zones adjacent to whale and wood falls and networks of canyons.

**Figure 5:**
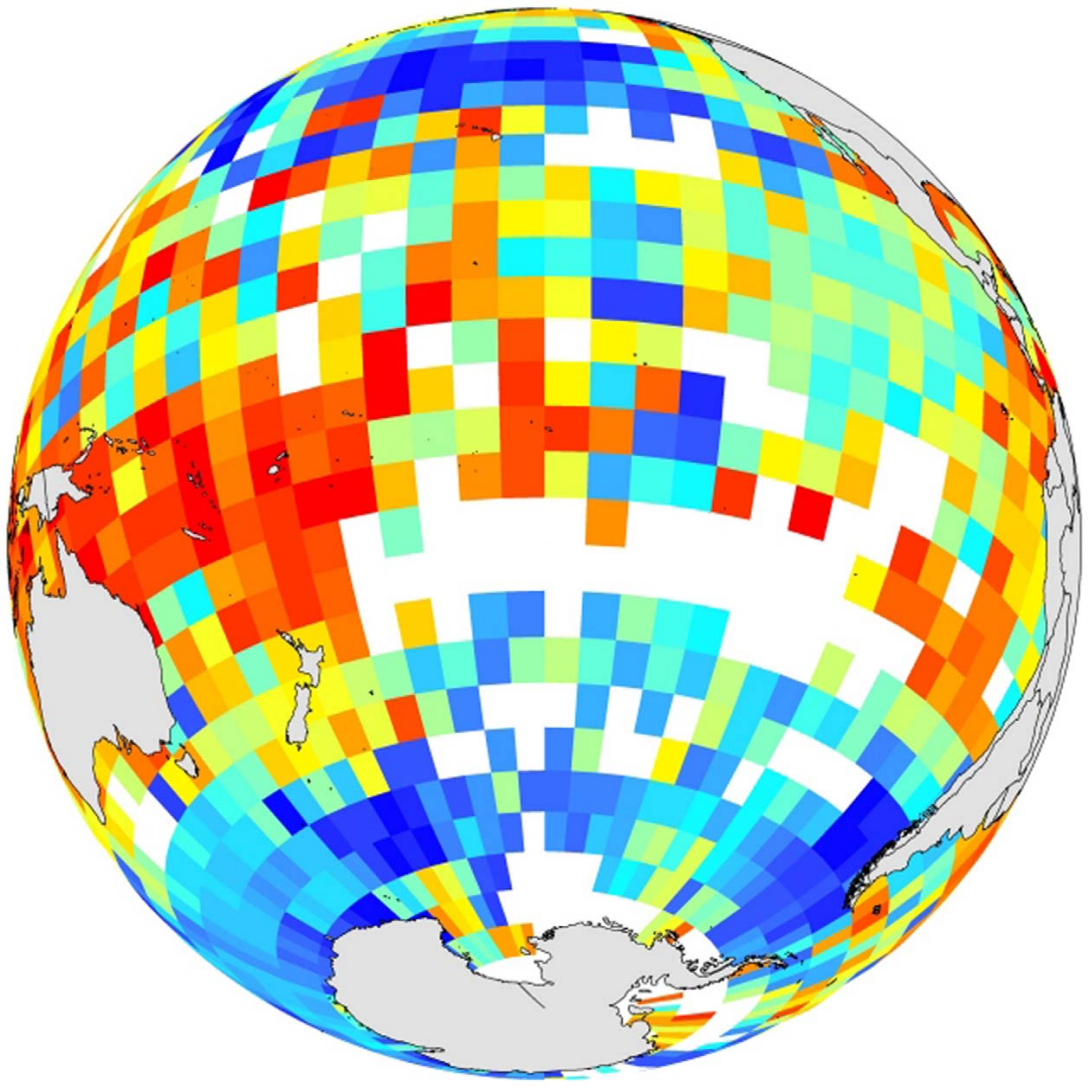
HFI* map (www.coml.org) showing how biodiversity “hot-spots” to West and East in the South Pacific are separated by areas devoid of data. *HFI: Hurlbert's First Index is a sample-size independent proxy for species richness. Here, colours (red = high) show predicted numbers of distinct species in a random sample of 50 observations; white: areas still awaiting collection of 50+ observations!

While the South Pacific provides connectivity between established biodiversity and evolutionary hotspots, in a range of habitat types, however, it also includes some of Earth's most poorly studied deep-ocean regions juxtaposed directly against those “hot-spots”. One of the most striking images from the synthesis activities of the Census of Marine Life (Figure 5) is a plot of Hurlberts First Index (HFI), a sample-size independent proxy for species richness, throughout Earth's Ocean Basins (www.coml.org). What is particularly notable in the projection shown, focused on the South Pacific, is that “hot-spot” areas of high biodiversity to the west and east are separated by areas where insufficient data exist to assign an HFI value because insufficient biological observations (50 or more) have yet to be conducted, throughout the entire history of deep-ocean research, within any of the “blank” boxes (pixel size: 5° Latitude×5° Longitude)! In this single ocean basin, all of the habitats present must share a common history and will have interacted, both biologically and geologically, throughout their formative history. What we recommend for a future and concerted effort, therefore, is an internationally coordinated project to investigate the interconnected deep-ocean habitats and ecosystems – from ocean margins to ridge-crests at 2000–3000 m depth, abyssal plains >3000 m deep and ocean trenches that lie more than 10,000 m below sea level – of the South Pacific Ocean: arguably Earth's largest and yet least understood deep-ocean basin. Specifically, we recommend a programme that would seek to address the following key questions:

1. How do species richness and community diversity vary across diverse habitat types, latitudes, depths and nutrient supply, both locally and regionally in the South Pacific?
2. Do the deep-ocean fauna of the South Pacific reveal patterns of connectivity via gene flow, or patterns of isolation and endemism to their various specific habitat types?
3. Is the vast oligotrophic abyss beneath the South Pacific gyre a biodiversity sink where food limitation exceeds adaptive limits, yielding a region of depauperate biodiversity?
4. Are the South Pacific abyssal, vent, seep and seamount fauna evolutionarily and functionally distinct from each other and from the fauna in other ocean basins?
5. Do the patterns of diversity and connectivity observed in South Pacific ecosystems render them particularly vulnerable to anthropogenic impact and/or variations in patterns of oceanic productivity that are predicted from climate change models?

**Figure 6:**
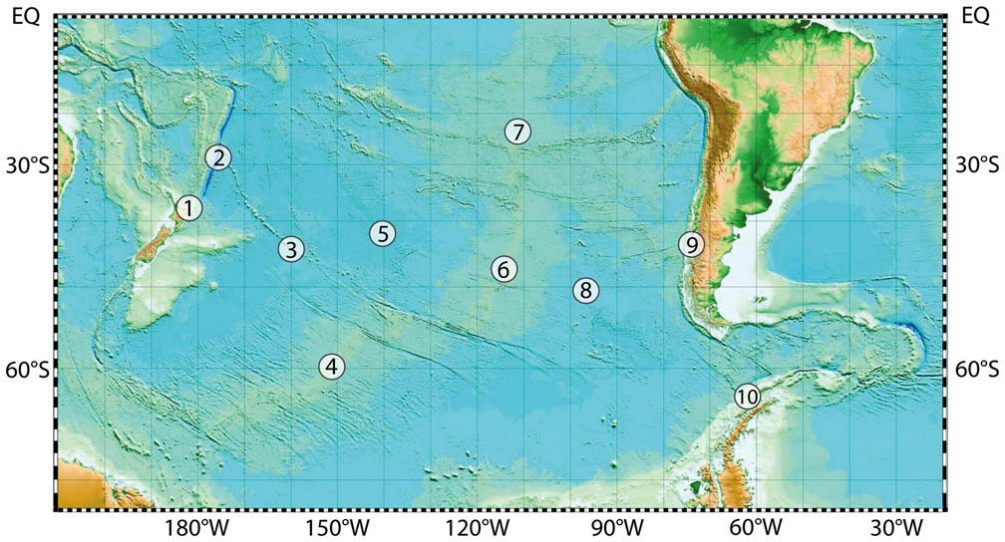
Bathymetric projection of the South Pacific Ocean with numbered field locations as described in the text. 1, Tonga-Kermadec arc; 2, deep-ocean trenches; (3) mid-plate seamounts of the Louisville Ridge; 4 & 6, Pacific-Antarctic Ridge; 5 & 8, abyssal plains; 7, Southern EPR; 9, Chile margin; 10, Bransfield Strait back-arc basin.

Our vision is that this programme could be implemented over a period of a decade in this single ocean basin, using international coordination to gain access to ships of multiple nationalities and the most sophisticated deep-submergence capabilities worldwide. Such a South Pacific programme would allow us to investigate a broad range of distinct ecosystems and obtain the samples required to characterise the intersection of inter-connected ecosystems and their seafloor habitats, from microbes to meio-, macro- and megafauna, at key abyssal plain, continental margin, vent, seep, whale and seamount sites located across the length and breadth of the South Pacific Ocean (Figure 6). Specific targets that could be selected from within this region include: the volcanically and tectonically active ocean margins of the Tonga-Kermadec arc (1) and Chile (9), where OMZs, seeps and vents exist in close proximity; (2) deep ocean trenches; (3) mid-plate seamounts of the Louisville Ridge, a hotspot chain that extends from New Zealand to the East Pacific Rise; abyssal plains (5, 8) that underlie some of the most oligotrophic open-ocean waters; and vents along the Pacific-Antarctic Ridge, north and south of the Polar Front (4,6), on the southern East Pacific Rise (Earth's fastest-spreading ridge) (7), and in the isolated Bransfield Strait back-arc basin (10). The international umbrella for efficient coordination of this work could readily be provided by the INDEEP initiative whose objectives are to promote research in the deep-ocean realm in the areas of: 1) Taxonomy and Evolution; 2) Biodiversity and Biogeography; 3) Population Connectivity; 4) Ecosystem Functioning; and 5) Anthropogenic Impact and Science Policy.

We believe that such a programme would have the potential to change, completely, the way we think about, study and train new scientists to understand diverse deep-ocean ecosystems. The project would provide key information for large-scale understanding of the ecology, biogeography, biodiversity inter-connectivity and evolution of the marine biodiversity of the deep South Pacific and allow direct comparisons with other oceans across comparable latitudes. Such a project would also have a strong exploratory component, providing for exciting new discoveries including, almost certainly, the identification and description of novel species and adaptations previously unknown to science. Although the scope and ambition of the project outlined here, across a largely uninvestigated ocean basin, may speak to some of purely academic “blue skies” research, we anticipate that the knowledge to be gained is also likely to be of critical importance for the development of the applied understanding that will be required for the future management and conservation of living and mineral resources in the deep ocean. Finally, the scale and ambition of the project outlined here would almost certainly lend itself to extensive, multi-media forms of outreach with the potential to expose millions of people to both the exciting expeditions and discoveries throughout the South Pacific and the concept of the common heritage of Earth's deep oceans.

In conclusion, the deep ocean has provided some of the most spectacular and paradigm-changing observations and data over the last 40 years. There is no doubt in our minds that the deep sea will continue to challenge scientists to devise ways and means of discovering and analysing its secrets into the future. Just as the discovery of hydrothermal vents could never have been made had scientists restricted their observations to remote sensing of the global mid-ocean ridge crest, it seems inevitable that even more exciting discoveries await as we extend our focus to the deepest seafloor abyssal plains and on, to the very depths of the deep-ocean trenches found at the far end of the plate tectonic cycle, associated with subduction zones.
